# Case Report: Structured MRI assessment of posterior thalamic infarction in a distribution compatible with posterior choroidal artery territory presenting as Déjerine-Roussy syndrome in an adolescent: differentiating arterial ischemia from venous thrombosis and thalamic neoplasm

**DOI:** 10.3389/fradi.2026.1869031

**Published:** 2026-06-12

**Authors:** ZainEdeen Zyadah, Hala Issa, Rana Issa, Anees Naser

**Affiliations:** 1 Faculty of Medicine, Al-Quds University, Jerusalem, Palestine; 2 Faculty of Medicine, Arab American University, Ramallah, Palestine; 3 Department of Neurology, Al-Makassed Hospital, Jerusalem, Palestine

**Keywords:** apparent diffusion coefficient, cerebral venous thrombosis, Déjerine-Roussy syndrome, diffusion-weighted imaging, pediatric arterial ischemic stroke, pharmacovigilance, posterior choroidal artery, posterior thalamic infarction

## Abstract

**Background:**

Isolated thalamic lesions with post-contrast T1 hyperintensity in children may mimic arterial infarction, cerebral venous thrombosis, inflammatory disease, or neoplasm. Structured interpretation of standard magnetic resonance imaging (MRI) sequences, particularly diffusion-weighted imaging/apparent diffusion coefficient (DWI/ADC) correlation and interval follow-up, can help distinguish these entities. Posterior thalamic infarction in a distribution compatible with posterior choroidal artery territory is rare in pediatric patients.

**Case Presentation:**

A previously healthy 15-year-old female developed headache and blurred vision on the day of tetanus-diphtheria (Td) vaccination, followed by progressive right hemibody numbness, impaired hand dexterity, and severe cold allodynia over 10 days. Examination localized to the left thalamus, consistent with Déjerine-Roussy syndrome. MRI on 2 June 2024, day 10 after symptom onset, showed DWI hyperintensity with subtle corresponding ADC reduction in the left posterior thalamic/pulvinar lesion, supporting early-subacute infarction. T2/fluid-attenuated inversion recovery (FLAIR) distribution was compatible with posterior choroidal artery territory involvement. Susceptibility-weighted imaging (SWI) showed no hemorrhage or venous-congestion pattern. Post-contrast MPRAGE demonstrated patchy T1 hyperintensity interpreted as probable enhancement, but true enhancement could not be definitively confirmed because corresponding pre-contrast T1-weighted images were unavailable. Etiologic workup was unrevealing, and the event was classified as cryptogenic pediatric arterial ischemic stroke (AIS). Follow-up MRI showed reduced post-contrast T1 hyperintensity and encephalomalacic evolution.

**Discussion:**

The unilateral territorial pattern, DWI/ADC abnormality, lack of hemorrhagic venous features, and follow-up maturation favored arterial infarction over venous thrombosis or neoplasm. Available post-contrast images showed no obvious filling defect of the major dural venous sinuses or principal deep venous structures, although dedicated magnetic resonance venography/computed tomography venography (MRV/CTV) was not performed. Corresponding pre-contrast T1 images were unavailable, limiting definitive enhancement assessment. The temporal association with Td vaccination is reported for pharmacovigilance; causality cannot be established from a single case.

**Conclusion:**

This case illustrates a rare adolescent posterior thalamic infarct presenting as Déjerine-Roussy syndrome and highlights the value of DWI/ADC and follow-up MRI in evaluating thalamic lesions with post-contrast T1 hyperintensity.

## 1 Introduction

Isolated thalamic lesions with post-contrast T1 hyperintensity on MRI pose a diagnostic challenge because the differential includes arterial infarction, cerebral venous thrombosis (CVT), inflammatory disease, and neoplasm. These entities differ substantially in management, making accurate clinical-radiological interpretation essential. The thalamus is a paired deep gray matter structure involved in sensory, motor, cognitive, arousal, and pain-processing networks (1, 2). Its arterial supply arises mainly from perforating branches of the posterior communicating and posterior cerebral arteries, classically grouped into tuberothalamic, paramedian, inferolateral/thalamogeniculate, and posterior choroidal territories (3–5). Posterior choroidal artery (PChA) infarction is uncommon and may involve the pulvinar, posterior thalamic nuclei, lateral geniculate body, hippocampus, and adjacent posterior temporal structures (3–7).

Pediatric AIS is rare but clinically important because of its potential for long-term morbidity (8, 9). Posterior circulation stroke represents a minority of pediatric AIS, and isolated posterior thalamic infarction is particularly uncommon (10–13). Pediatric AIS mechanisms include arteriopathy, cardiac disease, infection-related inflammation or thrombosis, thrombophilia, dissection, malignancy-associated mechanisms, and cryptogenic causes (8, 14–16). Déjerine-Roussy syndrome is a central post-stroke pain syndrome associated with thalamic injury and may present with contralateral sensory disturbance, allodynia, hyperalgesia, and persistent neuropathic pain (17–19).

According to the Palestinian Ministry of Health, the Td vaccine is administered in three doses over 14 years, beginning at 18 months of age, followed by booster doses at 6 and 15 years (20). The Td vaccine has a favorable safety profile, with systemic symptoms such as malaise, headache, and myalgia typically mild and self-limited (21, 22). Serious neurological events after tetanus-containing vaccines are rare. Brachial neuritis is recognized after tetanus-toxoid-containing vaccination (23), while large surveillance studies have not established a significant increase in major neurological conditions after Td or tetanus-diphtheria-acellular pertussis vaccination (24–26).

We report an adolescent with posterior thalamic infarction in a distribution compatible with PChA territory involvement, presenting as Déjerine-Roussy syndrome. The case illustrates the value of structured interpretation of standard MRI sequences, particularly DWI/ADC and follow-up MRI, in distinguishing infarction from CVT and neoplasm. The temporal association with Td vaccination is reported for pharmacovigilance only.

## 2 Case presentation

A previously healthy 15-year-old female presented to the emergency department 10 days after a school-administered Td vaccine. On the day of vaccination, she developed bitemporal headache and blurred vision, followed within 24 h by right hemibody numbness and impaired right-hand dexterity. Symptoms were intermittent over the ensuing week, but the sensory disturbance persisted. On day 10, she developed severe cold-evoked right hemibody pain, prompting emergency evaluation.

The patient was alert and hemodynamically stable, with blood pressure 100–106/70–76 mmHg, heart rate 87–95 beats per minute, and oxygen saturation 95%–98%. Cranial nerves were intact. Right hemiparesis was present, with Medical Research Council grade 4–4+/5 power in the upper limb and approximately 4/5 power in the lower limb. Pronator drift and an antalgic high-steppage gait were noted. Deep tendon reflexes were brisk on the right, with an extensor plantar response. Sensory examination revealed markedly reduced light touch and impaired proprioception over the right hemibody, with relative preservation of pinprick and prominent cold allodynia. This constellation localized the lesion to the left thalamus and adjacent efferent projections, consistent with Déjerine-Roussy thalamic pain syndrome (17). Cerebellar examination was normal.

Non-contrast computed tomography (CT) showed left thalamic hypoattenuation consistent with subacute ischemic injury. Computed tomography angiography (CTA) and magnetic resonance angiography (MRA) of the head and neck were reported to show patent major intracranial and cervicocephalic arteries without stenosis, aneurysm, vascular malformation, or dissection. Carotid duplex was unremarkable. Brain MRI on 2 June 2024, day 10 after symptom onset, showed DWI hyperintensity with subtle corresponding ADC reduction in the left pulvinar/posterior thalamus, supporting early-subacute infarction (Figures 1A,B). T2/FLAIR showed a confluent posterior thalamic lesion with mild retrolenticular extension, in a distribution compatible with PChA territory involvement (Figures 1C,D) (3–7). SWI showed no hemorrhage or venous-congestion pattern (Figure 1E). Post-contrast magnetization-prepared rapid gradient echo (MPRAGE) demonstrated patchy territorial T1 hyperintensity interpreted as probable enhancement, without nodular, ring-like, or mass-like morphology; however, true enhancement could not be definitively confirmed because corresponding pre-contrast T1-weighted images were unavailable (Figures 1F–H). No obvious filling defect of the major dural venous sinuses or principal deep venous structures was evident on the available post-contrast images; however, dedicated MRV/CTV was not performed.

**Figure 1:**
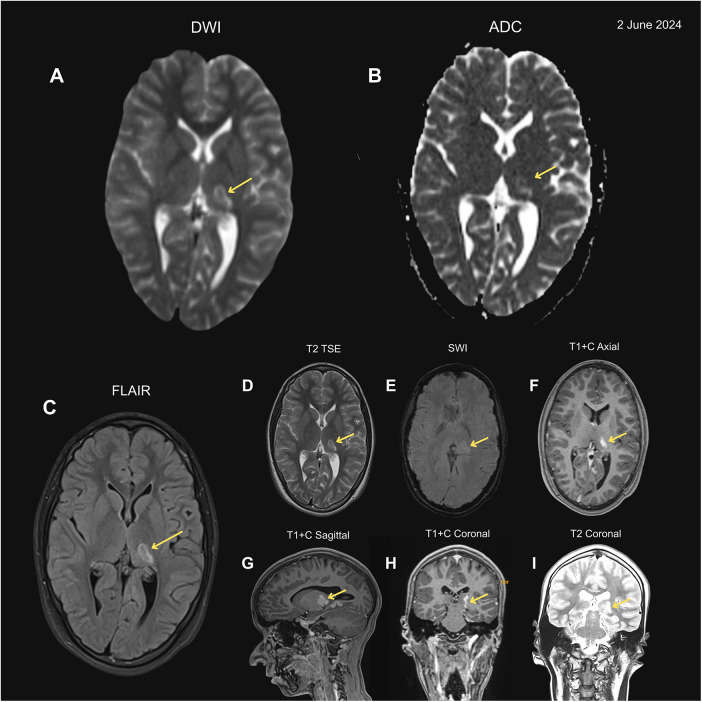
Initial brain MRI. MRI on 2 June 2024, day 10 after symptom onset. Yellow arrows indicate the left posterior thalamic/pulvinar lesion. **(A)** DWI shows left posterior thalamic/pulvinar hyperintensity. **(B)** ADC shows subtle corresponding reduction. **(C)** T2 FLAIR and **(D)** T2 TSE show a left posterior thalamic lesion with mild retrolenticular extension. **(E)** SWI shows no hemorrhage or venous-congestion pattern. **(F–H)** Post-contrast MPRAGE in axial, sagittal, and coronal planes shows patchy territorial T1 hyperintensity interpreted as probable enhancement, without mass-like morphology; true enhancement could not be definitively confirmed because corresponding pre-contrast T1-weighted images were unavailable. **(I)** Coronal T2 TSE shows the craniocaudal extent of the posterior thalamic lesion without midline shift or contralateral thalamic abnormality.

Inpatient cardiac monitoring and serial electrocardiograms (ECGs) excluded atrial fibrillation but identified an rSr′ morphology in lead V1 with normal QRS duration, consistent with an incomplete right bundle branch block pattern (Figure 2). Transthoracic echocardiography with agitated-saline bubble study on 4 June 2024 showed normal cardiac anatomy and biventricular function, with no right-to-left shunt at rest or during Valsalva. A 48-hour Holter monitor was negative for paroxysmal arrhythmia (Supplementary Report S1).

**Figure 2:**
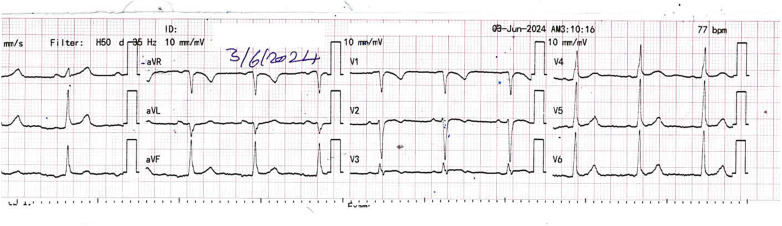
Electrocardiogram. Normal sinus rhythm with rSr′ morphology in V1 and QRS duration <120 ms, consistent with incomplete right bundle branch block.

Laboratory evaluation was unrevealing. Platelet counts, prothrombin time (PT), activated partial thromboplastin time (aPTT), and international normalized ratio (INR) were normal throughout admission and at three-month follow-up. Antithrombin activity was 103.4%. C-reactive protein (CRP) was 0.6 mg/L and erythrocyte sedimentation rate (ESR) was 13 mm/hr. Extended thrombophilia testing, including protein C, protein S, factor V activity, Factor V Leiden, and prothrombin G20210A genetic testing, was normal. Antiphospholipid antibody testing, including lupus anticoagulant, anticardiolipin IgG/IgM, and anti-beta-2-glycoprotein-1 IgG/IgM, was negative at admission and remained negative at 12 weeks; therefore, there was no laboratory evidence supporting antiphospholipid syndrome (APS). Antinuclear antibody (ANA) testing was negative.

Cerebrospinal fluid (CSF) analysis on 4 June 2024 showed clear fluid with red blood cell count 825 cells/µL and white blood cell count 2 cells/µL, consistent with a traumatic tap. Glucose was 84.2 mg/dL and protein was 27.15 mg/dL. CSF cytology showed no malignant cells. Cervical spine MRI excluded intramedullary pathology as an alternative cause of the hemibody deficits (Figure 3).

**Figure 3:**
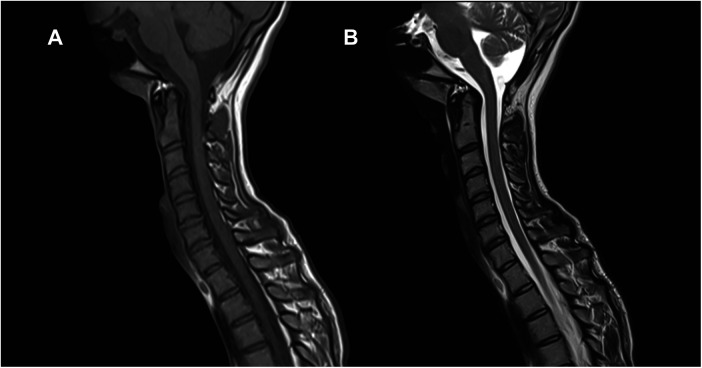
Normal cervical spine MRI. Sagittal **(A)** T1- and **(B)** T2-weighted images. Normal spinal cord morphology, caliber, and signal intensity throughout; no intramedullary lesion or structural abnormality. Excludes spinal cord pathology as an alternative etiology for the right hemibody deficits.

Empirical intravenous methylprednisolone was administered because of initial concern for inflammatory or neoplastic pathology. It was discontinued after the imaging pattern favored infarction and CSF cytology showed no malignant cells. Aspirin 100 mg daily was initiated for secondary prevention. Atorvastatin 40 mg daily was also started during admission; however, evidence for routine statin therapy in pediatric AIS is limited, and its use should be individualized (8, 14, 16). Motor strength improved substantially during admission, but hemibody sensory disturbance and central neuropathic pain persisted at discharge. Follow-up MRI on 3 August 2024 showed interval reduction in lesion size and post-contrast T1 hyperintensity, with evolving encephalomalacic change, strongly supporting infarct maturation and making untreated neoplasm unlikely (Figure 4). The patient was discharged with outpatient neurology follow-up and rehabilitation (Supplementary Table S1).

**Figure 4:**
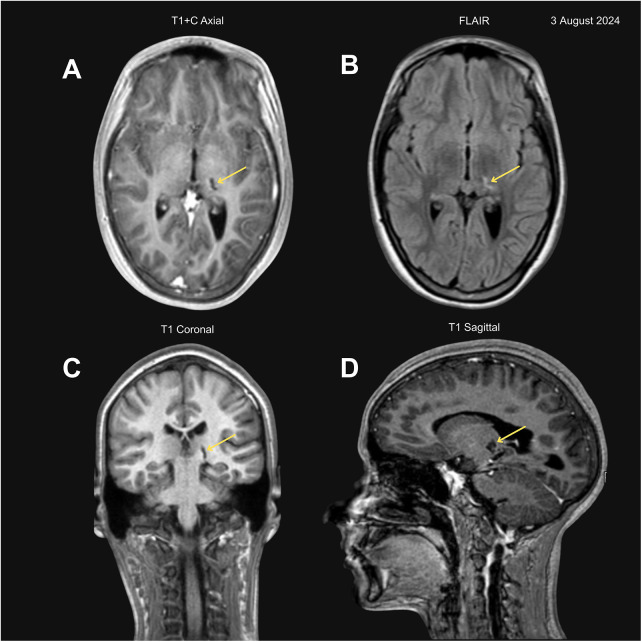
Follow-up brain MRI demonstrating subacute-to-chronic infarct evolution. MRI was obtained on 3 August 2024. Yellow arrows indicate the left posterior thalamic lesion site. **(A)** Axial post-contrast T1-weighted image shows interval reduction in post-contrast T1 hyperintensity at the lesion site. **(B)** Axial FLAIR shows interval edema resolution with evolving encephalomalacic change in the left posterior thalamus. **(C)** Coronal T1-weighted image demonstrates focal volume loss/encephalomalacic change in the left posterior thalamus with preserved contralateral thalamic morphology. **(D)** Sagittal T1-weighted image shows residual posterior thalamic signal/structural change without progressive mass effect, midline shift, or leptomeningeal T1 hyperintensity. Overall, the interval decreases in post-contrast T1 hyperintensity, resolution of edema, and development of focal volume loss support infarct maturation and make untreated neoplasm unlikely.

## 3 Discussion

### 3.1 Neuroimaging basis for differentiating arterial infarction from CVT and neoplasm

The main neuroradiological value of this case lies in the structured interpretation of an isolated posterior thalamic lesion with post-contrast T1 hyperintensity. The differential diagnosis included arterial ischemic infarction, CVT, inflammatory disease, and thalamic neoplasm. The final interpretation was supported by convergence of diffusion imaging, lesion topography, susceptibility imaging, post-contrast T1 pattern, clinical localization, and follow-up evolution.

1. **Arterial Infarction Versus Cerebral Venous Thrombosis:** DWI/ADC was central to the diagnosis. Brain MRI on 2 June 2024, day 10 after symptom onset, demonstrated DWI hyperintensity with subtle corresponding ADC reduction in the left posterior thalamic/pulvinar lesion. Given the subacute timing, the ADC reduction was interpreted as supportive of early-subacute ischemic injury rather than hyperacute infarction. The lesion's unilateral posterior thalamic distribution, clinical thalamic pain syndrome, and subsequent evolution toward encephalomalacia collectively favored arterial infarction. CVT was an important alternative diagnosis because deep venous thrombosis can involve the thalami and may mimic inflammatory or neoplastic lesions. However, CVT was considered less likely because the lesion was unilateral, territorially distributed, and lacked hemorrhagic venous features on SWI. Deep venous thrombosis more commonly produces bilateral thalamic edema and may be associated with hemorrhagic venous infarction or venous congestion. No obvious filling defect of the major dural venous sinuses or principal deep venous structures was evident on the available post-contrast images. Nevertheless, SWI does not directly prove venous patency, and dedicated MRV/CTV was not performed. Therefore, CVT was considered less likely based on the overall clinical-radiological pattern, while acknowledging that optimized venographic imaging was unavailable.
2. **Arterial Infarction Versus Thalamic Neoplasm:** Thalamic neoplasm was also considered because the lesion showed post-contrast T1 hyperintensity interpreted as probable enhancement. However, the lesion's territorial configuration, diffusion abnormality, mild mass effect relative to signal abnormality, and follow-up evolution toward reduced post-contrast T1 hyperintensity and encephalomalacic change favored infarct maturation. This pattern made untreated neoplasm unlikely, although true enhancement could not be definitively confirmed because corresponding pre-contrast T1 images were unavailable.

Overall, the imaging diagnosis did not rely on a single sequence or feature. Instead, DWI/ADC abnormality, posterior thalamic/pulvinar topography, absence of hemorrhagic venous features, non-mass-like post-contrast T1 hyperintensity, clinical thalamic localization, and interval encephalomalacic evolution collectively supported early-subacute arterial infarction in a distribution compatible with PChA territory involvement.

### 3.2 Clinical-anatomical correlation and Déjerine–Roussy syndrome

The patient's clinical syndrome was anatomically concordant with the imaging findings. Her contralateral sensory disturbance, cold allodynia, and persistent central neuropathic pain were compatible with disruption of thalamic somatosensory relay pathways, consistent with Déjerine-Roussy syndrome (17–19). Déjerine-Roussy syndrome classically follows thalamic injury and may manifest with sensory loss, allodynia, hyperalgesia, and persistent neuropathic pain (17–19). In this case, the posterior thalamic/pulvinar location provided a plausible substrate for the patient's dissociated sensory symptoms and cold-evoked hemibody pain. This clinical-imaging concordance strengthened localization to the left thalamus.

The patient also had mild right hemiparesis, pronator drift, brisk reflexes, and an extensor plantar response. These findings may reflect involvement of adjacent corticospinal projections or neighboring posterior limb/internal capsule fibers within the same regional ischemic process. Motor strength improved substantially during admission, whereas sensory disturbance and central pain persisted. Follow-up MRI demonstrated encephalomalacic change and focal volume loss in the left posterior thalamus. This irreversible structural injury may explain the persistence of central neuropathic pain despite motor improvement. Central post-stroke pain affects a minority of stroke survivors but is strongly associated with thalamic lesions (18, 19). Adult pharmacotherapy commonly includes agents such as amitriptyline and lamotrigine, while conventional analgesics are often ineffective (18). Pediatric-specific evidence remains limited, supporting individualized long-term follow-up.

### 3.3 Pediatric posterior thalamic infarction in a distribution compatible with posterior choroidal artery territory

This case adds to the limited pediatric literature on posterior thalamic infarction in a distribution compatible with PChA territory ischemia (3–13). Its distinctive features include adolescent presentation, Déjerine-Roussy syndrome, a thalamic lesion with post-contrast T1 hyperintensity mimicking alternative pathology, documented follow-up evolution, and temporal association with Td vaccination. The PChA territory can include the pulvinar, posterior and medial thalamic nuclei, lateral geniculate body, hippocampus, and adjacent posterior temporal structures (3–7). In this patient, the lesion was centered in the left posterior thalamus/pulvinar with mild retrolenticular extension, a distribution compatible with PChA territory involvement. Because small thalamic perforators cannot be directly mapped on the available vascular imaging, this territory assignment remains radiological rather than angiographically proven.

### 3.4 Temporal association with Td vaccination and pharmacovigilance considerations

The temporal association between Td vaccination and symptom onset is clinically notable and warrants pharmacovigilance reporting. However, temporal proximity alone cannot establish causality, particularly in a single case report.

Headache and blurred vision began on the day of vaccination, followed by progressive right hemibody symptoms over the next 10 days. This timing supports reporting the event as a temporal association. However, a negative etiologic workup does not establish vaccine causation. Therefore, Td vaccination is interpreted as a temporal pharmacovigilance signal rather than a definite or probable trigger.

Serious neurological events after tetanus-containing vaccines are rare. Brachial neuritis is recognized after tetanus-toxoid-containing vaccination (23), but epidemiological data do not establish a causal association between Td vaccination and pediatric AIS (27, 28). Based on the World Health Organization Adverse Events Following Immunization (WHO AEFI) framework, this event is most consistent with an indeterminate temporal association because the timing is compatible but definitive evidence that the vaccine caused the stroke is lacking (29).

### 3.5 Etiologic classification and secondary prevention

Despite vascular imaging, thrombophilia and antiphospholipid testing, CSF analysis, bubble echocardiography, cervical spine MRI, and 48-hour cardiac monitoring, no conventional etiology was identified. The event was therefore classified as cryptogenic pediatric AIS (8, 14, 16). The case is better classified as cryptogenic pediatric AIS than as embolic stroke of undetermined source (ESUS), an adult construct developed for non-lacunar cryptogenic infarction with suspected embolic mechanism.

In the absence of cardioembolic disease, dissection, or a defined hypercoagulable disorder, aspirin was selected for secondary prevention in accordance with pediatric AIS practice (8, 14, 16). Atorvastatin was started during admission, but evidence for routine statin therapy in pediatric AIS is limited and use should be individualized. Interval vascular imaging may be considered to evaluate for evolving focal cerebral arteriopathy, which can be occult on initial vascular imaging (8, 14, 16).

## 4 Limitations

Several limitations warrant acknowledgment. First, the temporal association with Td vaccination does not establish causality; no direct evidence of vaccine-mediated vasculitis, CSF inflammation, immune activation, or prothrombotic change was identified (27–29). Second, dedicated MRV/CTV was unavailable; therefore, venous assessment relied on available post-contrast images and indirect MRI features rather than optimized venographic imaging. Third, representative CTA/MRA images were unavailable for publication, although radiology reports described patent major arteries. Fourth, corresponding pre-contrast T1 images were unavailable, limiting direct confirmation of true enhancement. Fifth, perfusion MRI, spectroscopy, diffusion tensor imaging, and vessel-wall MRI were not performed. Sixth, anti-neutrophil cytoplasmic antibodies, anti-double-stranded DNA antibodies, and extended rhythm monitoring were not obtained.

## 5 Conclusion

We report an adolescent with posterior thalamic infarction in a distribution compatible with PChA territory involvement, presenting as Déjerine-Roussy syndrome. The diagnosis was supported by DWI/ADC abnormality, unilateral territorial morphology, absence of hemorrhagic venous features, absence of an obvious major venous filling defect on available post-contrast images, and follow-up encephalomalacic evolution. The case highlights the value of structured interpretation of standard MRI sequences, particularly DWI/ADC and follow-up MRI, in evaluating thalamic lesions with post-contrast T1 hyperintensity. The temporal association with Td vaccination warrants pharmacovigilance reporting but remains indeterminate and does not establish causality.

## Data Availability

The original contributions presented in the study are included in the article/Supplementary Material, further inquiries can be directed to the corresponding author.

## References

[1] Herrero MT Barcia C Navarro JM. Functional anatomy of thalamus and basal ganglia. Childs Nerv Syst. (2002) 18(8):386–404. 10.1007/s00381-002-0604-1 12192499

[2] Chen XY Wang Q Wang X Wong KS. Clinical features of thalamic stroke. Curr Treat Options Neurol. (2017) 19(2):5. 10.1007/s11940-017-0441-x 28251587

[3] Schmahmann JD. Vascular syndromes of the thalamus. Stroke. (2003) 34(9):2264–78. 10.1161/01.STR.0000087786.38997.9E 12933968

[4] Percheron G. The anatomy of the arterial supply of the human thalamus and its use for the interpretation of the thalamic vascular pathology. Z Neurol. (1973) 205(1):1–13. 10.1007/BF00315956 4126735

[5] Javed K Reddy V Das JM. “Neuroanatomy, posterior cerebral arteries”. In: StatPearls. Treasure Island, FL: StatPearls Publishing (2023) 30860709

[6] Gaillard F Sharma R Hacking C. Posterior Choroidal Artery Infarct. Radiopaedia.org. Melbourne, Australia: Radiopaedia Australia Pty Ltd (2008)

[7] Neau JP Bogousslavsky J. The syndrome of posterior choroidal artery territory infarction. Ann Neurol. (1996) 39(6):779–88. 10.1002/ana.410390614 8651650

[8] Sun LR Lynch JK. Advances in the diagnosis and treatment of pediatric arterial ischemic stroke. Neurotherapeutics. (2023) 20(3):633–54. 10.1007/s13311-023-01373-5 37072548 PMC10112833

[9] Oleske DM Cheng X Jeong A Arndt TJ. Pediatric acute ischemic stroke by age-group: a systematic review and meta-analysis of published studies and hospitalization records. Neuroepidemiology. (2021) 55(5):331–41. 10.1159/000518281 34464952 PMC8491514

[10] Mackay MT Prabhu SP Coleman L. Childhood posterior circulation arterial ischemic stroke. Stroke. (2010) 41(10):2201–9. 10.1161/STROKEAHA.110.583831 20829517

[11] Ganesan V Chong WK Cox TC Chawda SJ Prengler M Kirkham FJ. Posterior circulation stroke in childhood: risk factors and recurrence. Neurology. (2002) 59(10):1552–6. 10.1212/01.WNL.0000033092.87560.1A 12451196

[12] Carey S Wrogemann J Booth FA Rafay MF. Epidemiology, clinical presentation, and prognosis of posterior circulation ischemic stroke in children. Pediatr Neurol. (2017) 74:41–50. 10.1016/j.pediatrneurol.2017.05.007 28676245

[13] Fink M Slavova N Grunt S Perret E Regenyi M Steinlin M. Posterior arterial ischemic stroke in childhood. Stroke. (2019) 50(9):2329–35. 10.1161/STROKEAHA.119.025154 31345132

[14] Amlie-Lefond C. Evaluation and acute management of ischemic stroke in infants and children. Continuum (N Y). (2018) 24(1):150–70. 10.1212/CON.0000000000000559 29432241

[15] Asakai H Cardamone M Hutchinson D Stojanovski B Galati J Cheung M. Arterial ischemic stroke in children with cardiac disease. Neurology. (2015) 85(23):2053–9. 10.1212/WNL.0000000000002036 26408496 PMC4676756

[16] Rawanduzy CA Earl E Mayer G Lucke-Wold B. Pediatric stroke: a review of common etiologies and management strategies. Biomedicines. (2023) 11(1):2. 10.3390/biomedicines11010002 PMC9856134 36672510

[17] Jahngir MU Qureshi AI. “Déjerine-Roussy syndrome”. In: StatPearls. Treasure Island, FL: StatPearls Publishing (2023) 30085589

[18] Klit H Finnerup NB Jensen TS. Central post-stroke pain: clinical characteristics, pathophysiology, and management. Lancet Neurol. (2009) 8(9):857–68. 10.1016/S1474-4422(09)70176-0 19679277

[19] Liampas A Velidakis N Georgiou T Vadalouca A Varrassi G Hadjigeorgiou GM. Prevalence and management challenges in central post-stroke neuropathic pain: a systematic review and meta-analysis. Adv Ther. (2020) 37(7):3278–91. 10.1007/s12325-020-01388-w 32451951 PMC7467424

[20] Palestinian Ministry of Health. National Immunization Programme Schedule. Ramallah: Palestinian Ministry of Health (2023).

[21] Choi J Choo EJ Huh A Choi S Eom JS Lee JS. Immunogenicity and safety of diphtheria-tetanus vaccine in adults. J Korean Med Sci. (2010) 25(12):1727–32. 10.3346/jkms.2010.25.12.1727 21165286 PMC2995225

[22] Thierry-Carstensen B Jordan K Uhlving HH Dalby T Sorensen C Jensen AM. A randomised, double-blind, non-inferiority clinical trial on the safety and immunogenicity of a tetanus, diphtheria and monocomponent acellular pertussis vaccine in comparison to a tetanus and diphtheria vaccine when given as booster vaccinations to healthy adults. Vaccine. (2012) 30(37):5464–71. 10.1016/j.vaccine.2012.06.073 22776216

[23] Liang JL Tiwari T Moro P Messonnier NE Reingold A Sawyer M. Prevention of pertussis, tetanus, and diphtheria with vaccines in the United States: recommendations of the Advisory Committee on Immunization Practices. MMWR Recomm Rep. (2018) 67(2):1–44. 10.15585/mmwr.rr6702a1 29702631 PMC5919600

[24] Jackson ML Yu O Nelson JC Nordin JD Tartof SY Klein NP. Safety of repeated doses of tetanus toxoid, reduced diphtheria toxoid, and acellular pertussis vaccine in adults and adolescents. Pharmacoepidemiol Drug Saf. (2018) 27(8):921–5. 10.1002/pds.4569 29862604 PMC6541919

[25] Tseng HF Sy LS Qian L Marcy SM Jackson LA Glanz J. Safety of a tetanus-diphtheria-acellular pertussis vaccine when used off-label in an elderly population. Clin Infect Dis. (2012) 56(3):315–21. 10.1093/cid/cis871 23196953

[26] Jackson LA Yu O Belongia EA Hambidge SJ Nelson J Baxter R. Frequency of medically attended adverse events following tetanus and diphtheria toxoid vaccine in adolescents and young adults: a Vaccine Safety Datalink study. BMC Infect Dis. (2009) 9(165). 10.1186/1471-2334-9-165 PMC2765445 19804643

[27] Cho J Park J Jo H Yim Y Woo HG Oh J. Global estimates of reported vaccine-associated ischemic stroke for 1969–2023: a comprehensive analysis of the World Health Organization global pharmacovigilance database. J Stroke. (2024) 26(3):463–7. 10.5853/jos.2024.01536 39266013 PMC11471359

[28] Fullerton HJ Hills NK Elkind MS Dowling MM Wintermark M Glaser CA. Infection, vaccination, and childhood arterial ischemic stroke: results of the VIPS study. Neurology. (2015) 85(17):1459–66. 10.1212/WNL.0000000000002065 26423434 PMC4631070

[29] World Health Organization. Causality Assessment of an Adverse Event Following Immunization: User Manual for the Revised wHO Classification, 2nd Edn. Geneva: World Health Organization (2019).

