# Supplementary material for: Case Report: Structured MRI assessment of posterior thalamic infarction in a distribution compatible with posterior choroidal artery territory presenting as Déjerine-Roussy syndrome in an adolescent: differentiating arterial ischemia from venous thrombosis and thalamic neoplasm
Source: Front Radiol. 2026 Jun 12;6:1869031. doi: 10.3389/fradi.2026.1869031 (PMC13303791; doi:10.3389/fradi.2026.1869031)
Supplement: Supplementary file 1 [file Table1.docx]

**Supplementary Table 1. Clinical and Imaging Timeline.**

| **Day** | **Date** | **Clinical Event** |
| --- | --- | --- |
| **Day 0** | **23 May 2024** | Td vaccination; onset of bitemporal headache and blurred vision. |
| **Day 1** | **24 May 2024** | Development of right hemibody numbness and impaired right-hand dexterity. |
| **Day 10** | **2 June 2024** | Severe cold-evoked right hemibody pain prompted emergency department presentation. Brain MRI with DWI/ADC demonstrated left posterior thalamic/pulvinar diffusion abnormality. |
| **Day 12** | **4 June 2024** | Transthoracic echocardiography with bubble study and CSF analysis were performed. |
| **Day 72** | **3 August 2024** | Follow-up MRI showed reduced post-contrast T1 hyperintensity and encephalomalacic evolution. |
