# Supplementary material for: Case Report: Structured MRI assessment of posterior thalamic infarction in a distribution compatible with posterior choroidal artery territory presenting as Déjerine-Roussy syndrome in an adolescent: differentiating arterial ischemia from venous thrombosis and thalamic neoplasm
Source: Front Radiol. 2026 Jun 12;6:1869031. doi: 10.3389/fradi.2026.1869031 (PMC13303791; doi:10.3389/fradi.2026.1869031)
Supplement: Supplementary file 2 [file Datasheet1.pdf]

[redacted]

Name: [redacted]
Sex:
File No
Ward:

Transthoracic Echocardiography Report

[redacted] [redacted]

Patient's weight: [redacted] **Date & Time of procedure:** 04/06/2024 - 09:37

1.Echocardiography at rest.

Dimensions	cm	Normal	Dimensions	cm	Normal
**Left Ventricle**			Left Atrium/Volume	2	2.7-4.0 cm / 16-28 ml/m2
**Diastole**	3.6	3.9-5.6 cm	E-Wave Velocity	100	60-80 cm/s
**Systole**	2	2.0-4.1 cm	A-Wave Velocity	95	20-35 cm/s
**Septum (D)**	0.6	0.6-1.0 cm	E/A-Ratio	1.2	0.8-2/ age-dependent
**Septum (S)**	0.8	0.8-2.0 cm	E/e'-Ratio septal	-	<8
**Posterior Wall (D)**	0.6	06-1.0 cm	Diastolic Dys-function	-	
**Ejection Fraction**	65	> 55 %	Aortic max. Velocity	125	100-220 cm/s
**Ascending Aorta**	2.1	2.4-3.6 cm	Pulmonary max. Velocity	130	80-140 cm/s
**Right Ventricle/mid RV**	2.1	1.9-3.5 cm	Tricuspid Reg. Velocity	-	< 280 cm/s
**Inferior V. Cava**	-	< 2.1 cm	TAPSE	-	> 1.7 cm

**Left Atrium:**	Normal size.
**Left Ventricle:**	Normal size and good function.
**Right Ventricle:**	Normal size and function.
**Aortic Valve:**	Trileaflet valve.<br>Normal mobility and function.
**Mitral Valve:**	Good mobility and function.<br>No regurgitation.
**Tricuspid Valve:**	Good function with trace regurgitation.
**Pulmonary Valve:**	Well visualized without dysfunction.
**Pericardium/Shunt:**	No effusion. No signs of shunt.

Notes/ Recommendation

Bubbles test done no shunts.
[redacted]

[redacted]
